# Salicylic Acid Binding Proteins (SABPs): The Hidden Forefront of Salicylic Acid Signalling

**DOI:** 10.3390/ijms20184377

**Published:** 2019-09-06

**Authors:** Igor Pokotylo, Volodymyr Kravets, Eric Ruelland

**Affiliations:** 1 V.P. Kukhar Institute of Bioorganic Chemistry and Petrochemistry, National Academy of Sciences of Ukraine, 02094 Kyiv, Ukraine; 2 Institut d’Ecologie et des Sciences de l’Environnement de Paris, Université Paris-Est, UPEC, 94010 Créteil, France; 3 Institut d’Ecologie et des Sciences de l’Environnement de Paris, CNRS, UMR 7618, 94010 Créteil, France

**Keywords:** Salicylic acid, salicylic acid binding protein, SABP, NPR1, stress response, pathogens

## Abstract

Salicylic acid (SA) is a phytohormone that plays important roles in many aspects of plant life, notably in plant defenses against pathogens. Key mechanisms of SA signal transduction pathways have now been uncovered. Even though details are still missing, we understand how SA production is regulated and which molecular machinery is implicated in the control of downstream transcriptional responses. The NPR1 pathway has been described to play the main role in SA transduction. However, the mode of SA perception is unclear. NPR1 protein has been shown to bind SA. Nevertheless, NPR1 action requires upstream regulatory events (such as a change in cell redox status). Besides, a number of SA-induced responses are independent from NPR1. This shows that there is more than one way for plants to perceive SA. Indeed, multiple SA-binding proteins of contrasting structures and functions have now been identified. Yet, all of these proteins can be considered as candidate SA receptors and might have a role in multinodal (decentralized) SA input. This phenomenon is unprecedented for other plant hormones and is a point of discussion of this review.

## 1. Introduction

Salicylic acid (SA, 2-hydroxybenzoic acid) is a phenolic plant hormone. SA has a well- documented role in plant responses to environmental stresses including chilling [1], freezing [2,3], heat [4], heavy metals [5], salt [6], drought [7] and, notably, reactions to biotrophic pathogens [8]. Indeed, SA has a major role in plant innate immunity and systemic acquired resistance (SAR)—a whole-plant resistance triggered by a local infection [9]. SA also plays a role in the regulation of stomatal closure [10] and seed germination [11], among others. Despite these beneficial effects, constitutive over-accumulation of SA stunts plant growth [12,13].

**Figure 1:**
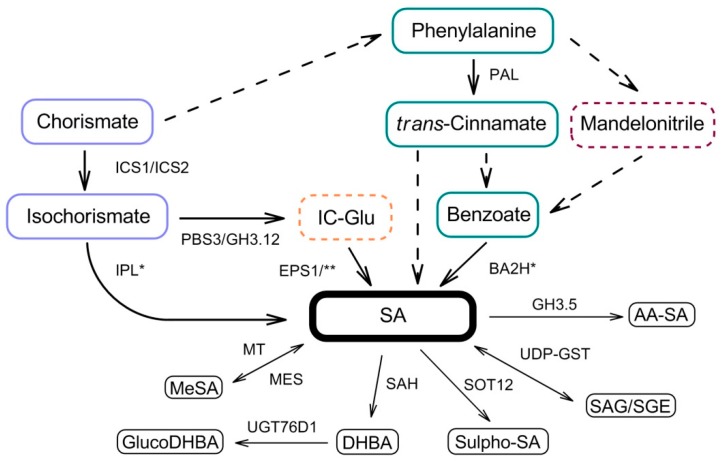
SA biosynthesis pathways in plants. Solid arrows represent single enzymatic steps. Dashed arrows represent multiple consecutive enzymatic steps. AA-SA, amino acid-SA conjugate; BA2H, benzoic acid 2-hydroxylase; DHBA, dihydroxybenzoic acid; EPS1, Enhanced Pseudomonas Susceptibility 1; GH3.5, Gretchen Hagen 3.5; ICS, isochorismate synthase; IC-Glu, isochorismate-9-glutamate; IPL, isochorismate pyruvate lyase; MeSA, methyl-salicylate; MES, methyl-salicylate esterase; MT, metyl transferase; PAL, phenylalanine ammonia-lyase; PBS3, avrPphB susceptible 3; SAG, salicylic acid glucoside; SAH, salicylic acid hydroxylase; SGE, salicylic acid glucose ester; SOT12, sulfotransferase 12; UDP-GST, UDP-glycosyltransferase; UGT76D1, UDP-glycosyltransferase 76D1; *, enzyme not cloned in plants; **, non-enzymatic decay.

SA is accumulated in plants following stress exposure. In *Arabidopsis thaliana sid2* mutants, SA accumulation induced by *Pseudomonas syringae* pv. *tomato* (Pst) DC3000 expressing *avrRpt2* was abolished [14]. These plants were characterized as isochorismate synthase 1 (ICS1)-deficient [15]. ICS1 is a component of SA biosynthesis pathways (Figure 1). The second ICS-coding gene (*ICS2*) provides a much lower contribution to SA synthesis in Arabidopsis. Both ICS1 and ICS2 proteins are found in chloroplasts [16]. Suppression of the barley *HvICS* gene by RNAi led to an impaired SA accumulation induced by *Fusarium graminearum* infection [17]. Recently, ICS1 was suggested to be post-translationally activated via direct interaction with PHB3—a member of the prohibitin protein family in Arabidopsis [18]. Following the production of isochorismate by ICS, it is believed to be converted to SA by isochorismate pyruvate lyase (IPL). However, the coding gene has not yet been cloned in plants. Recently, an alternative route for the processing of isochorismate was suggested. It could be converted to isochorismate-9-glutamate by avrPphB Susceptible 3 (PBS3, also known as Gretchen Hagen 3.12, GH3.12); isochorismate-9-glutamate would lead to SA either via a passive decay [19] or by Enhanced Pseudomonas Susceptibility 1 (EPS1)—an enzyme with isochorismoyl-glutamate A pyruvoyl-glutamate lyase activity [20] (Figure 1). Interestingly, the activity of GH3.12/PBS3 is inhibited by SA in vitro (see below).

Another pathway for SA synthesis involves the phenylalanine ammonia lyase (PAL) converting phenylalanine (Phe) to *trans*-cinnamic acid. This pathway occurs in the cytosol. Four PAL-encoding genes are found in *A. thaliana* [21]. The PAL pathway was shown to be active in poplar infected with *Botryosphaeria dothidea* fungus [22]. Yet, another route for SA biosynthesis involving mandelonitrile, a Phe derivative, was proposed to be functional in peach plants (Figure 1). Mandelonitrile-treated peach plants accumulated more SA and were more resistant to *Plum pox virus* [23]. The synthesis of SA through this pathway occurs through a benzoate intermediate. The exact role of mandelonitrile in SA accumulation is still a matter of discussion.

SA can be converted to a number of derivatives. These molecules have roles as either transportable forms of SA (such as methyl-salicylate, MeSA) or inactive/storage forms of SA (Figure 1). Among the latter are glucosylated SA derivatives—salicylic acid glucoside (SAG) and salicylic acid glucose ester (SGE) [24]. These molecules are stored in the vacuole and can be reversely converted to SA [25]. Hydroxylated SA derivatives—2,3-dihydroxybenzoic acid (DHBA) and 2,5-DHBA—are catabolic SA forms in the cytosol [26]. Yet, glycosylation of these molecules by UGT76D1 (an UDP-glycosyltransferase) was shown to be a part of the immune response to Pst DC3000 in *A. thaliana* and regulated ICS1-dependent SA production [27]. In *A. thaliana*, SA can also be sulfonated by cytosolic SOT12 sulphotransferase. SA sulfonation has a similar positive feedback on SA accumulation in stressed plants [28]. Aspartyl amino acid conjugates of SA are thought to attenuate SA signaling, and they are formed by GH3.5 in *A. thaliana* [29].

Biotic stress-induced SA accumulation is controlled by three protein regulators: Enhanced Disease Susceptibility 1 (EDS1), Phytoalexin Deficient 4 (PAD4) and Senescence Associated Gene 101 (SAG101) [30]. It is known that EDS1 binds at least several TIR-NB-LRR receptors (used by the plant to sense the presence of pathogens) [31] and interacts with either PAD4 or SAG101 [32]. EDS1, PAD4, and SAG101 are all lipase/esterase-like proteins. However, the exact mode of their input to SA synthesis is yet to be established and does not necessarily rely on these enzymatic activities. Recently, an EDS1-PAD4 complex was found to inhibit MYC2 [33], which is a positive regulator of jasmonic acid (JA) pathway signaling that itself antagonizes SA (for review see Ref. [34]). This is thought to be an evolution-selected mechanism to block the action of coronatine—a JA-mimicking compound produced by the bacteria to subdue SA-driven defenses. EDS1 and PAD4 could also have a role independent of SA. For instance, the activation of *FLAVIN-DEPENDENT MONOOXYGENASE1* (*FMO1*) expression in Arabidopsis in response to thaxtomin A (a bacterial toxin) was dependent of EDS1 and PAD4 but independent of ICS1-generated SA [35].

Basal SA accumulation is controlled by multiple inputs. In many SA-overaccumulating mutants reviewed in Ref. [13], the connection between the function of the protein encoded by the altered gene and SA pathway signaling is vague. In rice, the knocking-down of *SEC3A*—coding for an exocyst protein complex component that guides exocytic vesicles to the plasma membrane—led to SA over-accumulation [36]. The *pi4kβ1β2* double mutant, altered in two phosphatidylinositol-4-kinases, also accumulates high SA levels, but the reason for this is still being investigated [12].

After SA is produced, it interacts with Nonexpressor of Pathogenesis-related protein 1 (NPR1)—a key transcriptional regulator of SA signaling. SA affects NPR1 in at least two ways: i. NPR1 directly binds SA [37]; and ii. SA induces redox changes in the cell that conditions NPR1 monomerization [38]. Monomeric NPR1 shuttles to nuclei where it interacts with TGA transcription factors leading to the expression of *PATHOGENESIS-RELATED* (*PR*) genes involved in the set-up of plant immune defenses [13]. The regulation of PR gene transcription by NPR1 has been shown to involve histone acetylation via CBP/p300-family histone acetyltransferases [39].

Intriguingly, the mechanisms of SA perception are not fully understood. For instance, some signaling events are triggered upstream of NPR1 monomerization and/or shuttling into the nucleus. This is the case of the cell redox change, but also of phosphatidylinositol 4-kinase (PI4K) and phospholipase D (PLD) activation [40,41,42]. Moreover, some responses to SA are observed in NPR1-deficient mutants [43,44,45]. Therefore, NPR1 cannot be the only SA-binding protein (SABP) in plants. NPR1 paralogs, NPR2 [46], NPR3 and NPR4 [47,48], also bind SA. Moreover, high-throughput biochemical screens have provided a list of almost 100 candidate SABPs [49,50]. Many of these proteins are important enzymes of primary carbon metabolism. This challenges the classical paradigm of hormonal signaling where a ligand is recognized by a single/few receptor(s). Are these SABPs real SA receptors or are they false positive of high through-put omics techniques? If they really bind SA, what are the roles of such binding in SA-signaling pathways? And why do plant cells have so many SABPs. The role(s) of such a multitude of SABPs is still unexplained and offers a new paradigm for hormonal regulation in plants.

## 2. SA Binding Proteins

The multiplicity of SABPs has already been highlighted as an intriguing phenomenon [51]. For many of the SABPs, the physiological outcome of their interaction with SA is unclear. Moreover, it is not established whether and how the SABPs are integrated into the conventional NPR1 pathway. At least several plant SABPs have their animal orthologs that also bind SA [51]. In animals, SA and its derivatives (e.g., aspirin) act not as hormones but as therapeutic compounds that target proteins and prevent their role as disease components. How animals have developed such sophisticated responses to substances originating from plants is an interesting question by itself. Nevertheless, this suggests that SA can clearly act in a NPR1-free environment [51]. Here, we present an up-to-date vision of these problems, characterise the canonical and newly identified plant SABPs, and discuss how they can be integrated into a unified SA-signaling network.

### 2.1. SABP1—Catalase

SABP1 was purified from tobacco as a soluble cytosolic protein that binds SA with a *K*_d_ of 14 μM [52]. *SABP1* was cloned and the resulting 57 kDa protein was characterized as catalase [53]. Its activity was drastically inhibited by SA but not by inactive SA analogues in vitro [54]. This effect corresponds well to the results obtained using tobacco cell suspensions where SA could inhibit the total catalase activity [55].

In *A. thaliana*, catalase 2 (CAT2) shares 78% sequence identity with tobacco SABP1 (UniProt P49319). SA inhibited CAT2 catalase activity in vitro and total catalase activity in leaves of SA-pre-treated plants. However, the direct binding of SA to CAT2 has not been tested [56]. CAT2 was demonstrated to have a role in SA-mediated resistance to a biotrophic pathogen (Pst DC3000) by inhibiting indole-3-actic acid (IAA, an auxin) and JA accumulation [56]. Inhibition of CAT2 by SA leads to a H_2_O_2_ increase in plants upon pathogen infection. This promotes sulfenylation (sulfenic acid conjugation) of tryptophan synthetase b subunit1 (TSB1) at Cys308 leading to the inhibition of its activity. This enzyme acts in the IAA biosynthesis pathway and as a result, SA antagonizes IAA accumulation. In parallel, SA diminished the stimulatory effect of CAT2 protein on the in-vitro activity of acyl-CoA oxidases (ACX2/ACX3) implicated in jasmonic acid (JA) biosynthesis. It is not clear if this effect is due to the catalase activity of CAT2. A direct interaction between CAT2 and ACX2/ACX3 has been observed. This interaction was impaired in SA-pre-treated plants [56].

Unlike wild-type (WT) plants, *sid2* mutants accumulated JA in response to Pst DC3000 (reflecting the antagonism of SA against JA accumulation). Such JA accumulation was diminished in *sid2cat2* double mutants, suggesting that CAT2 is indeed a positive regulator of JA production and thus plays a part in the SA-JA antagonism in plants [56]. The *cat1* (78% identity to SABP1) and *cat3* (76% identity to tobacco SABP1) Arabidopsis mutants were not altered in the biotic stress-induction of neither IAA nor JA accumulation [56].

The way SA binds SABP1 or catalase orthologues, from a molecular point of view, has not been deciphered.

### 2.2. SABP2—MeSA Esterase

SABP2 was similarly purified from tobacco and had a much higher affinity to SA (*K*_d_ = 90 nM) when compared to SABP1 [57]. A corresponding ORF encoding a 260-residue α/β fold hydrolase superfamily protein with a calculated molecular mass of 29 kDa was cloned and characterized. Its lipase activity towards *para*-nitrophenyl palmitate (measured by the release of *p*-nitrophenol) was drastically stimulated by SA in vitro. The silencing of SABP2 using the RNAi technique resulted in the lowering of SA-induced *PR1* expression and resistance level against *Tobacco mosaic virus* (TMV) [58]. Later, it was established that SABP2 activity was that of a methyl salicylate (MeSA) esterase, converting MeSA into SA, an end-product inhibitor of such activity. The co-crystallization of SABP2 with SA revealed that it is positioned carboxylate group-first in the active site [59]. The inhibition of methyl esterase (MES) activity of SABP2 by SA could be a mechanism to fine-tune active SA concentration in the cell.

SABP2 can also convert acibenzolar-S-methyl (a functional analogue of SA) into acibenzolar. Silencing of SABP2 in tobacco results in the loss of an acibenzolar-S-methyl effect on the induction of *PR1* expression and the onset of SAR [60].

MeSA (produced by salicylate carboxyl methyltransferases) is inactive but is more hydrophobic than SA and it easily penetrates cell membranes. Therefore, MeSA is, in line with other molecules such as pipecolic acid [61], considered to be a mobile signal of the SAR. Produced in infected cells, MeSA reaches distant leaves by phloem transport. In these so-called systemic tissues, it is converted into active SA via MES activity. The SA thus produced triggers-preventive defense responses in these distal leaves. SABP2 was established as a key enzyme of SAR in tobacco [62,63]. In potato, an orthologue of SABP2, StMES1, has been cloned. The enzymatic activity of StMES1 was inhibited by SA in vitro and its role was, in a similar way, linked to SAR development [64].

Two methyl esterase-encoding genes were identified in poplar [65]. In *A. thaliana*, 18 SABP2 orthologues were identified and at least five proteins (AtMES1,-2,-4,-7,and -9) were shown to possess an esterase activity acting on MeSA that was inhibited by SA in vitro [66]. In these two species, MES is also an important component of SAR [22,66].

### 2.3. SABP3—β Carbonic Anhydrase

SABP3 was identified in tobacco chloroplasts as β carbonic anhydrase (βCA). SABP3 binds SA with moderate affinity (*K*_d_ = 3.7 μM) [67]. CAs are ubiquitous and evolutionary-conserved enzymes that catalyse the interconversion of CO_2_ and bicarbonate (HCO_3_^−^). CAs have roles in photosynthesis, respiration, stomata movements, and lipid biosynthesis among others [68]. From an immunity point of view, SABP3 was required for the hypersensitive response (HR) in tobacco leaves infiltrated with *A. tumefaciens* expressing *Pto*:*avrPto* (R-avr gene pair). Two recombinant tobacco proteins—SABP3/βCA1 and βCA2—were shown to bind SA but not 4-hydroxybenzoic acid (4-HBA, an inactive isomer). Based on the fact that SABP3 could complement the phenotype of an oxidative stress-sensitive strain of *Saccharomyces cerevisiae* [67], it was suggested that SABP3 might have antioxidative properties.

In *A. thaliana*, the affinity of AtSABP3/βCA1 (a SABP3 orthologue) to SA and its CA activity is diminished in the presence of S-nitrosoglutathione (GSNO), a NO donor. These effects were not observed in a C280S AtSABP3-mutated protein, suggesting that they are due to the nitrosylation of Cys280 [69]. AtSABP3 knockout mutants have their resistance compromised towards Pst DC3000 (*avrB*). The resistance phenotype was restored when complemented by AtSABP3 but not by C280S AtSABP3 [69]. In this manner, AtSABP3 nitrosylation is clearly a requirement for its role in immunity. In contrast, the role of the binding of SA to AtSABP3 requires further research.

Indeed, there are contrasting data about SA influence on CA activity in plants. SA did not affect the CA activity of purified SABP3 from tobacco chloroplasts at physiological concentrations. The inhibition only occurred at concentrations as high as 3 mM, while binding occurred at lower concentrations [67]. A significant increase in CA activity was reported in leaves of SA-treated peppermint (*Mentha piperita*) [70]. In contrast, in *A. thaliana*, CA activity was reported to decrease following treatment with SA or with benzothiadiazole (a functional SA analogue), or after inoculation with *P. syringae* [71].

SABP3 orthologs from Arabidopsis and *Chenopodium quinoa* were found to physically interact with HCPro—a viral protein of *Turnip mosaic virus* (TuMV). This protein has RNA silencing suppressor (RSS) activity and counteracts host’s anti-viral RNA interference. The transient expression of HCPro antagonized AtSABP3 transcripts and protein accumulation in Arabidopsis [72]. AtSABP3 was thus established as a component of anti-viral defense. Whether this role of AtSABP3 is dependent on SA binding is still unknown. Unexpectedly, both AtSABP3-knockout and AtSABP3-overexpressing lines of Arabidopsis were compromised for their resistance to TuMV [72].

AtSABP3 bearing no signal peptide was shown to interact with NPR1 in a yeast two-hybrid assay. Intriguingly, this result was observed only when growth plates were supplemented with SA (but not with inactive isomer 4-HBA), suggesting that this interaction was SA dependent [71]. An AtSABP3-NPR1 interaction was also demonstrated in tobacco leaves in planta. Bimolecular fluorescence complementation revealed that this interaction occurred in the nucleus and the perinuclear region. The transient expression of GFP-NPR1 and MBP-AtSABP3 constructs led to co-purification of GFP-NPR1 together with MBP-AtSABP3 on amylose resin [71]. In the same study, interactions with NPR1 and NRB4 (a protein that is perhaps involved in SA perception), were similarly reported for at least several other cloned fragments/alternative splice variants of proteins representing the βCA family in Arabidopsis. These data suggest that βCA family members could be a part of SA signalling. However, an Arabidopsis *βca1* mutant deficient in AtSABP3 (or quintuple *βca1,2,3,4,6* mutant for that matter) was only partially insensitive to exogenous SA as suggested by pathogen resistance and *PR1* expression responses. The homozygous mutation in *βCA5* could not be tested due to plants sterility [71].

Note that amino acid residues required for SA binding to SABP3 and orthologues have not yet been identified.

### 2.4. NPR1/2/3/4—Signalling Proteins

NPR1 binds SA with high affinity in vitro (*K*_d_ = 140 nM) [37]. SA binding to NPR1 is Cu^2+^-dependent, implicates Cys521 and Cys529, and results in conformational changes of NPR1—a mechanism that could stand behind its role as a transcription cofactor with TGA [37].

NPR1 exists in the cytosol as an oligomer due to disulphide bridges between Cys82 and Cys216 of different subunits. In order to shuttle to the nucleus and act on regulating gene expression, NPR1 has to be monomerized [38]. This monomerization requires the reduction of the disulphide bonds. SA binding has been shown to facilitate de-oligomerization of recombinant NPR1. Yet, SA alone is not sufficient to trigger NPR1 monomerization [37]. The upstream SA-driven redox events that allow the reduction of the disulphide bonds in NPR1 are not fully understood. Two cytosolic thioredoxins, TRX-h3 and TRX-h5, have been shown to interact with NPR1 since they were pulled-down by the immobilised His-tagged N-terminal part of NPR1. Furthermore, co-immunoprecipitation of TRX-h5 with a NPR1-TAP fusion protein was stimulated by SA [73]. Thioredoxins are redox regulators and can reduce disulphide bridges [74,75]. Co-incubation of cell lysates with recombinant TRX-h5 led to an increase in NPR1-GFP monomers, thus suggesting that TRX-h5 plays a part in NPR1 monomerization. Both *trx-h3* and *trx-h5* mutants were compromised in *PR1* induction by exogenous SA [73]. In contrast, NPR1 monomerization is negatively regulated by S-nitrosylation at Cys156 [73].

In Arabidopsis, NPR3, NPR4 (both having 39% identity to NPR1) [48] and NPR2 (62% identity to NPR1) [46] are paralogues of NPR1. They have all been shown to bind SA. The affinities of NPR3 (*K*_d_ = 176nM) and of NPR4 (*K*_d_ = 23nM) to SA are known [76]. NPR3 [77], NPR4 [78] and NPR2 [46], similarly to NPR1, act in the nucleus. Interestingly, NPR3 and NPR4 were demonstrated to bind NPR1 in a SA-dependent manner in vitro. Based on the fact that the ability of cullin 3 (CUL3, a component of protein E3 ligase complex) to pull-down NPR1-GFP was reduced in a *npr3 npr4* genetic background, it was suggested that NPR3 and NPR4 act as adaptors for SA-dependent proteasome-mediated degradation of NPR1 [48]. In a similar manner, NPR3 and NPR4 were suggested to mediate the proteasome-dependent degradation of EDS1 [79].

NPR3 and NPR4 have been shown to have roles as transcriptional co-repressors that function in parallel to NPR1. They act as negative regulators of immunity. *SARD1* and *WRKY70* genes are, for instance, under the negative transcriptional control of NPR3/NPR4 [76]. Co-transformation of Arabidopsis protoplasts expressing a luciferase reporter gene under the control of either SARD1 or WRKY70 promoters with plasmids overexpressing either NPR3 or NPR4 resulted in the inhibition of luciferase expression. This effect was diminished when TGA-binding motifs were mutated in either SARD1 or WRKY70 promoter regions. SARD1 and WRKY70 expression were also partly de-repressed in TGA2/TGA5/TGA6-deficient plants. This suggests that TGA transcription factors are implicated in the negative control of their expression by NPR3 or NPR4. More importantly, SA could antagonise the observed transcriptional repression activity of NPR4 [76].

In potato, the StNPR3L (NPR3-like protein) was shown to interact with the transcription factor StbZIP61 and inhibit its transcriptional activation activity in an SA-dependent manner [80]. This could have a role in the regulation of SA accumulation in infected plants since the expression of *StICS1*, a SA biosynthesis gene, positively correlated to *StbZIP61* expression as suggested by RNAi and mutant studies [80].

NPR2 physically interacts with NPR1 in vitro, and in planta the overexpression of NPR2 could partly complement the *NPR1*-deficient phenotype [46]. The role of SA binding in this interaction is unknown.

### 2.5. Glutathione S-transferase

Several *A. thaliana* glutathione *S*-transferase (GST) isoenzymes (GSTF2, GSTF8, GSTF10, GSTF11) have been shown to bind SA [81]. The enzymatic activity of GSTF10, GSTF11 and that of GSTF8 (to a lesser extent) were inhibited by SA in vitro. GSTs comprise a large group of enzymes that catalyse at least several reactions in connection to glutathione conjugation. Promoters of many GST-encoding genes contain disease-related W-boxes and WT-box cis-regulatory elements. GSTs are a part of plant immunity with roles in glucosinolate (antimicrobial compound) metabolism and detoxification of mycotoxins among others [82].

### 2.6. Thioredoxins

The chloroplastic thioredoxin-m1 (TRXm1) was shown to bind SA in a high-throughput screen and later confirmed by surface plasmon resonance (SPR) analysis [50]. Reduced thioredoxins can reduce disulphides of target proteins [74,75]. The reduction of TRX arises either from NADPH through NADPH thioredoxin reductases or from ferredoxin via ferredoxin thioredoxin reductases. The effect of SA binding on TRXm1 activity has not been yet established. As mentioned earlier, the activity of other thioredoxins, the cytosol located TRX-h3 and TRX-h5, is required for NPR1 monomerization [73]. It could be interesting to investigate whether these cytosolic TRXs are also SA-binding proteins.

### 2.7. GH3—Acyl Acid Amido Synthetase

GH3.12/PBS3 from *A. thaliana* conjugates specific amino acids to acyl substrates (e.g., 4-substituted benzoates) in an Mg^2+^- and ATP-dependent manner. This enzyme binds SA in a ternary complex with AMP as shown by its crystallographic structure [83]. Due to the observed position in the active pocket, SA was suggested not to be a substrate but an inhibitor of AtGH3.12/PBS3. This is consistent with the fact that SA indeed inhibits AtGH3.12/PBS3 activity in vitro [84]. Conjugation of amino acids to plant hormones, such as jasmonic acid or auxins, is a common strategy aimed at controlling their active level in plant cells [85]. PBS3 was also shown to be an important enzyme of SA biosynthesis [19,20]. The role of AtGH3.12/PBS3 in SA signalling is, however, unclear. The *P. syringae*-induced free SA accumulation in *pbs3* mutants was actually higher than that of WT plants, but it was accompanied by the reduced pathogen resistance and retarded *PR1* expression in such plants. These effects were accompanied by the drastic diminution of SA-*O*-*β*-glucoside (SAG) accumulation [86]. The connection between SAG production and AtGH3.12/PBS3 has not been established yet.

Recently, PBS3 was shown to directly interact with EDS1—one of the three key protein regulators of SA pathway signalling (see above). In tobacco leaves, the interaction occurred both in the nucleus and cytoplasm as revealed by bimolecular fluorescence complementation assays. No interaction was observed for PBS3 and PAD4 or SAG101. Using an inhibitor approach, authors concluded that PBS3 could control EDS1 abundance in a post-translational manner by preventing its proteasome degradation—most likely by the 26S proteasome [79]. The role of SA binding to PBS3 was not investigated in this context.

It was reported that another GH3 family member, AtGH3.5, could accept SA as a substrate. In AtGH3.5-overexpressing Arabidopsis lines, a significant increase in the content of SA-aspartyl was registered [29]. The very same enzyme also produces inactive aspartyl conjugates of indole-3-acetic acid (IAA)—an auxin hormone. In this manner, AtGH3.5 could play a part in SA-IAA crosstalk.

### 2.8. GAPDH

Several isoforms (subunits) of *A. thaliana* GAPDH (glyceraldehyde 3-phosphate dehydrogenase)—GAPA-1, GAPA-2, GAPC-1, and GAPC-2—bind SA as demonstrated using SPR [87]. Among the detected SA-binding GAPDH isoforms, some are cytosolic (GAPC-1, GAPC-2) while others are plastidial (GAPA-1, GAPA-2) enzymes. They play essential roles in glycolysis (cytosolic) and Calvin cycle (plastidial), respectively. However, the effect of SA binding on GAPDH function is unknown.

Some GAPDH isoenzymes have been shown to be multifunctional. AtGAPC-1 is required for *Tomato bushy stunt virus* (TBSV) asymmetric replication via its direct association with a negative RNA strand of the virus. The binding of SA to AtGAPC-1 inhibits the association of the enzyme to the virus RNA. This was suggested to be a part of plant anti-viral defenses [87].

Interestingly, human GAPDH also binds SA and is similarly implicated in the regulation of the replication of some viruses [88]. SA binding suppresses HsGAPDH translocation to the nucleus. Whether a similar mechanism affecting GAPDH localization is employed in plants is yet to be established.

### 2.9. Alpha-ketoglutarate Dehydrogenase—Krebs Cycle Enzyme

The E2 subunit of the α-ketoglutarate dehydrogenase (αKGDE2) enzyme complex acts in the tricarboxylic acid (Krebs) cycle in mitochondria. This protein was shown to bind SA in two independent assays (photoaffinity labelling and SPR) both in Arabidopsis [81] and tomato [89]. The αKGDE2 activity was reduced almost by half in isolated mitochondria sampled from tomato leaves pre-treated with SA for 24 h. This could be due to either a transcriptional or translational regulation by SA and not to a direct effect of the binding of SA on the protein. The silencing of αKGDE2 resulted in the increase of tomato resistance to TMV. Interestingly, the treatment with SA could similarly induce resistance to TMV in WT plants but it could not enhance the resistance phenotype of αKGDE2-silenced plants [89]. Based on these facts, a suppression of αKGDE2 by SA was suggested to be a part of plant antivirus defenses.

### 2.10. Thimet Oligopeptidases + TPPII Exopeptidase—Proteolysis

In *A. thaliana*, there are three thimet oligopeptidases (TOP, zinc-dependent metalloendopeptidases) [90]. Two of them, TOP1 and TOP2, bind SA [49]. SA inhibited peptidase activities of TOP1 and, to a lesser extent, of TOP2, in vitro. For TOP1, kinetics indicated a non-competitive mechanism. SA treatment also inhibited the bulk peptidase activity in plant extracts as measured by the release of a fluorescent peptide marker. TOP1 contains a signal peptide and TOP1–GFP was found to be localized in chloroplasts. The inhibitory effect of SA on a truncated form of TOP1, lacking 110 N–terminal residues spanning the signal peptide, was much weaker. This suggests that SA could selectively affect TOP1 activity based on its localization. Based on mutant studies, both TOP1 and TOP2 were required for plant response to either Pst *avrRpt2* or Pst *avrRps4*. However, when tested with Pst *avrRpm1*, Pst *avrPphB* or *Pseudomonas syringae* pv. *maculicola* (Psm), no differences to WT plants were observed. Moreover, the assessed level of programmed cell death was actually lower in *top1-3* mutants inoculated with Pst avrRpt2 compared to WT [49].

TOP1 and TOP2 were found to produce homo- and heterodimers. The formation of TOP2-TOP2 and TOP1-TOP2 dimers were diminished by the addition of SA in isolated *A. thaliana* protoplasts. Authors suggested that the effect of SA could be due to redox changes since the effect of dithiothreitol, a strong reductant, led to a strong shift towards the presence of TOP1 and TOP2 monomeric forms in vitro [91]. The functional role of TOP dimers is unclear.

Another enzyme implicated in proteolysis that binds SA is tripeptidyl peptidase II [50].

### 2.11. MORC Proteins—Epigenetic Regulation

Microrchidia (MORC) proteins comprise a group of peculiar DNA-binding enzymes with ATPase, endonuclease and topoisomerase activities. These proteins can be potentially involved in epigenetic gene silencing [92]. In tomato, SlMORC1 binds SA as demonstrated by SPR analysis [93]. This interaction resulted in altered activities of SlMORC1 in vitro: SA suppressed ATPase and decatenation activities but not the DNA relaxation activity of SlMORC1.

### 2.12. HMGB3—DAMP Protein

High Mobility Group Box 3 (HMGB3) was shown to bind SA using SPR analysis [94]. The binding affinity of this protein to the immobilized 3-aminoethyl SA was very high (*K*_d_ = 1.5 nM). This protein was found to be a DAMP (damage-associated molecular pattern molecules) acting via BAK1 and BKK1 receptor kinases. Exogenous application of purified recombinant HMGB3 induced plant immune responses and was enough to improve Arabidopsis resistance to *B. cinerea*. HMGB3, when applied together with 1 μM SA, lost its effectiveness as a DAMP.

## 3. Response of SABPs to Treatments Linked to SA/Biotic Stress

**Figure 2:**
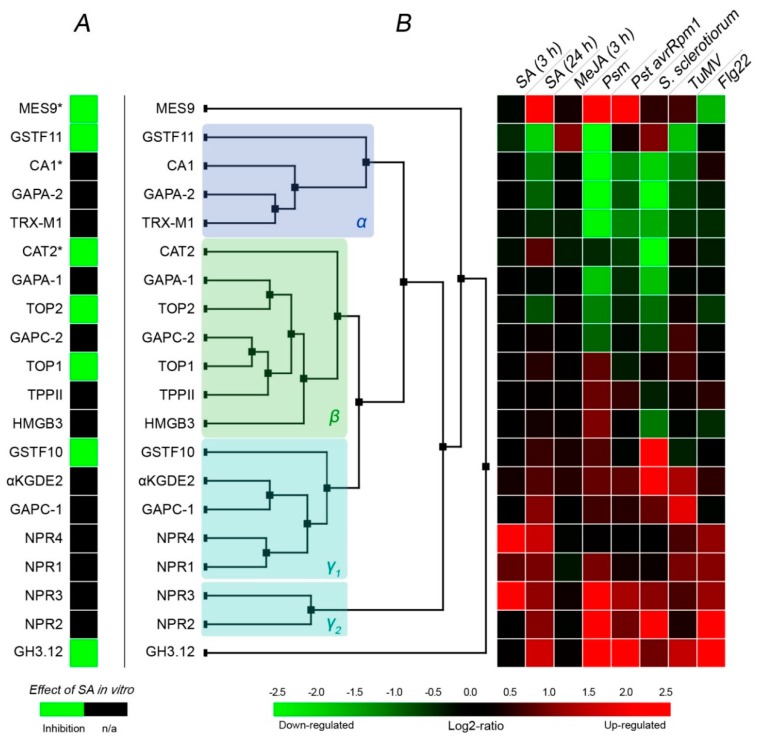
Effect of SA on the in-vitro catalytic activity of SABPs from *A. thaliana* (**A**) and transcriptional responses of corresponding coding genes (**B**). *, effect observed in tobacco orthologs. Transcriptomic data was mined using Genevestigator [95]. Experiment IDs: SA 3 h, AT-00113; SA 24 h, AT-00320; MeJA 3 h, AT-00110; Psm, AT-00406; Pst avrRpm1, AT-00106; *S. sclerotiorum*, AT-00681; TuMV, AT-00324; flg22, AT-00392. Note that the hierarchical clustering was performed on more experiments than the one used for the heat map (see the main text).

To sum up the above sections and to find common regulatory patterns, if any, among SABPs, we mined transcriptomics data for genes encoding the SABPs described above (Figure 2). In panel A, we show the effect of SA on the protein, when it is known. In our list are proteins from *A. thaliana*. However, the effects of SA on CAT2, MES9 and βCA1 were extrapolated from those observed for tobacco orthologs (SABP1, SABP2 and SABP3, respectively). SA treatment led to an inhibition of enzymatic activity of a number of SABPs in vitro. The activity of NtSABP3 was not affected by SA while the effect of SA on transcriptomic activities of NPR proteins was not considered. No example of SA activating a SABP enzymatic activity in vitro is currently available to us.

In panel B (Figure 2), we show two sets of transcriptomics data. A first set was used to draw the dendrogram showing the hierarchical clustering of SABPs based on their expression across 111 conditions. In these experiments, *A. thaliana* plants were challenged with bacterial (*Pseudomonas* spp.), fungal (*Sclerotinia sclerotiorum*, *Golovinomyces orontii*, *Hyaloperonospora arabidopsidis*) and viral (TuMV) pathogens. All of these conditions should directly implicate SA signaling responses. We could separate three clusters. The alpha cluster represents the genes whose expression was mostly inhibited by the treatments; the gamma clusters represent genes whose expression was induced by the treatments while the beta cluster represents genes exhibiting an intermediate situation.

In the second set of transcriptomics data (heat map), we illustrate how the expression of SABPs change when different aspects of SA signalling are stimulated. Here we included the SA treatment per se, but also treatments with methyl jasmonate (MeJa, a SA-antagonist hormone), model pathogens, and a flg22 elicitor—a fragment of flagellin, a protein from bacterial flagellum that triggers immunity responses. *NPR3* and *NPR4* are two genes early stimulated by SA (3 h). After a longer time (24 h), SA could both stimulate (*NRP* genes, *GH3.12*) and inhibit (*GSTF11*, *CA1*, *GAPA-2*) the expression of SABPs. No SABPs were reactive to MeJa. We could not observe significant differences in expression patterns of SABPs following treatments with Pst and Psm bacteria—the latter bearing the *avrRMP1* avirulence gene that activates effector-triggered immunity (ETI). The transcriptional responses to *S. sclerotiorum* fungus and TuMV were a tad divergent. In virus-treated plants, a negative effect on *GAPA-2*, *TRX-m1* and *CA1* expression was relieved. The same can be said for plants treated with flg22. It should be noted that across all modelled infections, the stimulation of *NPR* genes and *GH3.12* was quite consistent.

Therefore, similarly to what was observed in the clustering analysis, binding to SA concerns proteins that are both positively and negatively expressed in response to elicitation related to biotic stresses. All the SABP-coding genes behaved differently, but at least some SABPs are well synchronised at the transcriptomic level. For instance, NPR proteins, especially NPR2 and NPR3, appear to cluster together (gamma2), thus suggesting that they are involved in the same signaling cascade. Indeed, NPR2, NPR3 and NPR4 have been shown to interact physically with NPR1 [46,48]. The same transcriptomic connections are true for βCA1, GAPA-1 and TRXm1 in the alpha cluster, and KGDE2 with GAPC1 in the gamma1 cluster. How these connections at the transcriptomic level translate into interplay between protein functions is yet to be established.

It is also interesting to note that for GAPDH family members, some appear to be strongly inhibited in responses to immunity-related stresses (such as the plastidial GAPA2) while others are induced (such as cytosolic GAPC1). These enzymes are likely not to have the same role in SA-signaling pathways. Besides, there is no strong correlation between the effect of SA on the protein (panel A) and the way their genes react to immunity-related inputs (panel B): Proteins that are inhibited by SA are found in alpha and gamma clusters.

Interestingly, a human GAPDH gene is commonly used as a “housekeeping” reference gene in quantitative RT-PCR analyses [96]. The data presented in this paper, however, show that the same cannot be translated to GAPDH isogenes in Arabidopsis since a strong transcriptomic reaction to either viral (GAPC-1) or bacterial/fungal infections (GAPA-2) was observed.

## 4. Molecular Mechanisms of SA-Protein Interactions

**Figure 3:**
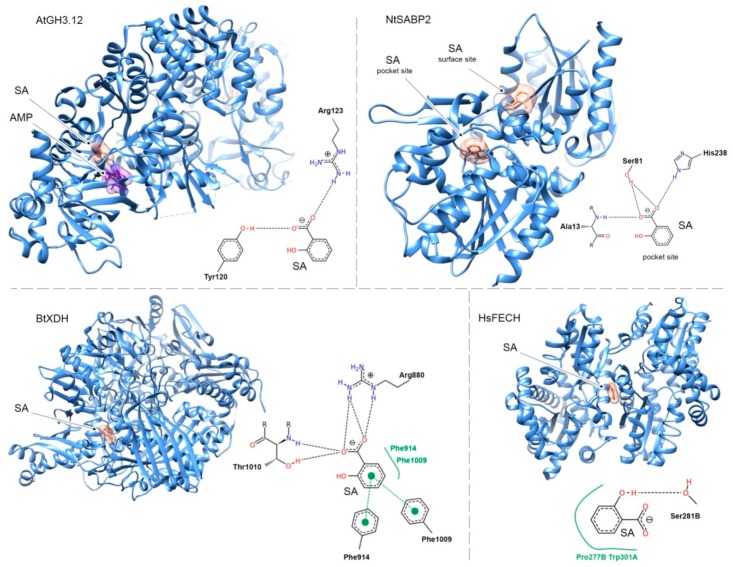
Molecular interactions between SA and selected SABPs. Black dashed links represent hydrogen bonds. Green dashed lines represent pi-pi stacking. Green solid lines represent hydrophobic interactions. Molecular graphic images were produced using UCSF Chimera [97].

Some plant SABPs have been crystallized with SA. Since no conserved SA binding motif is known, the analysis of crystallography data could help to better understand how binding occurs. Here we have focused on two plant proteins, AtGH3.12 and NtSABP2 (Figure 3).

In AtGH3.12, SA binding occurs in the active site. The carboxyl group of SA forms hydrogen bonds with side chains of Arg123 and Tyr120. Using the UCSF Chimera software [97], we could identify that SA also forms contacts (likely nonpolar interactions) with Gly326 and the side chain of Ile217 (Figure S1). While SA was co-crystallized with AMP in the active site of AtGH3.12, there is no direct interaction between SA and this cofactor (Figure S1).

For NtSABP2, two SA-binding sites have been found; an inner pocket, in the active site and a surface pocket that has been suggested to be a crystallographic artefact [59]. In the inner pocket, the carboxyl group of SA forms hydrogen bonds with Ala13, Ser81 and His238 residues (Figure 3). An interaction between the SA carboxyl group carbon chain and the carbon chain of Ser81 was predicted using UCSF Chimera (Figure S1). As for the surface-binding pocket, the carboxyl group of SA forms a hydrogen bond with Lys159, while interactions were predicted with Lys159, Leu132 and His158. As for the latter, the aromatic ring of SA is in a parallel plane to the imidazole ring of His158 (Figure S1).

Interestingly, many animal and human proteins have been co-crystallized with SA (or, alternatively, with acetyl SA—an aspirin). Similar mechanisms are apparently involved in the binding. Bovine milk xanthine dehydrogenase binds SA via hydrogen bonds formed between Thr1010, Arg880 and the carboxyl group of SA. Moreover, clear π–π stacking is formed between the aromatic ring of SA and aromatic rings of Phe914 and Phe1009 (Figure 3). In human ferrochelatase (FECH), however, it is a side hydroxyl group, and not the carboxylic group, that forms a hydrogen bond to Ser281 (Figure 3). In this protein (a homodimer), SA binds directly at the dimer interface, implicating hydrophobic amino acid residues from both monomers (Figure S1). It is worth mentioning that SA inhibits FECH activity in vitro. This could be due to induced conformational changes since the gel filtration elution profile was altered in the presence of SA [98].

Interestingly, for NPR1, SA binding was suggested to involve Cys521 and Cys529 based on protein mutation studies and requires the presence of Cu^2+^ ions. However, NPR1 has not yet been crystallized with SA [37].

In conclusion, SA binds to proteins both in plants and animals using similar mechanisms. As a common rule, binding occurs by hydrogen bond formation between the carboxyl group of SA and side chains of various amino acid residues (Arg, Tyr, Ala, Ser, His). Such interaction could be strengthened by nonpolar/π-π contacts made by the aromatic ring of SA. The side hydroxyl group of SA can also be involved. The change in its relative position is enough to alter the binding affinity of SA/4-HBA to proteins [81,89].

Many of the SABPs were identified by SPR analysis, a method where an immobilized 3-animoethyl SA is used as a ligand. This implies that interactions should occur on the surface of the proteins, at least in the SABPs identified with this technique.

## 5. Discussion and Conclusions

**Figure 4:**
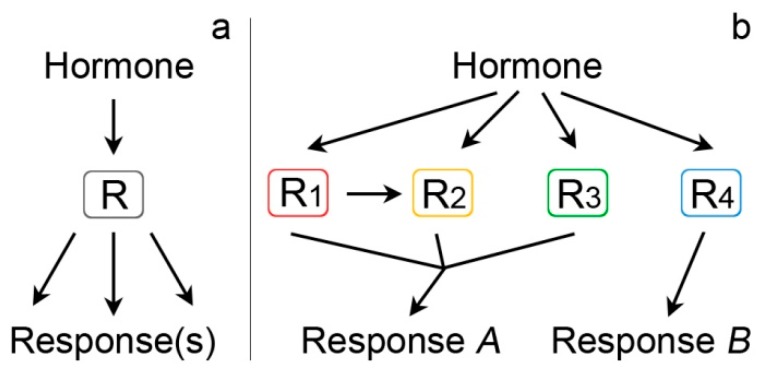
Model of conventional hormonal input via a single receptor (R) leading to downstream responses (**a**); model of SA multinodal input via multiple SABPs acting as independent receptors (**b**). Some receptors in model B may be in a functional connection (e.g., a putative SA-binding TRX acting on NPR1) and act in a single reception pathway.

SA interacts with multiple plant proteins. These include the canonical SA receptor, NPR1, and its paralogs, but also many other proteins with diverse roles in cell regulation. The role of these interactions is still not known. NPR1 is, without a doubt, a bottleneck of SA signaling, since in NPR1-deficient plants, SA-induced responses, e.g., the expression of PR genes [99], are drastically altered. The goal of this review was to give a physiological reasoning for SA binding to other plant proteins as a mode of multinodal input (Figure 4). In a canonical situation (panel A), a hormone binds a receptor (one protein or proteins of the same family) and activates a signaling cascade, leading to cell responses. This is true for many plant hormones (e.g., auxins and jasmonates). Based on available data, the SA-signaling pathway appears to be different (panel B). A number of structurally unrelated receptors exist in parallel. Each receptor is a node that will lead either to the same cascade, acting synergistically, or activate separate cascades.

This model is based on the fact that many SABPs (acting as potential SA receptors) are important enzymes where, at least for some of them, SA binding results in a modification of their activity in vitro (Figure 2). In most, if not all examples available to us where SA inhibits enzymes, SA is likely to obstruct substrate–enzyme interactions or take the place of a necessary cofactor.

SABPs are often connected to plant immunity. For instance, the silencing of αKGDE2 (a SABP) led to an increase in plant-virus resistance [89]. However, since many SABPs are crucial enzymes of basal metabolism, knocking them out could result in unspecific growth aberrations. Instead, point mutations that preserve enzymatic function but disrupt SA interactions should be introduced in planta to test the role of SA binding. Finding SA interaction sites in proteins with no crystallization data available is an intriguing task. This approach would also help to validate SA binding to SABPs in planta.

**Figure 5:**
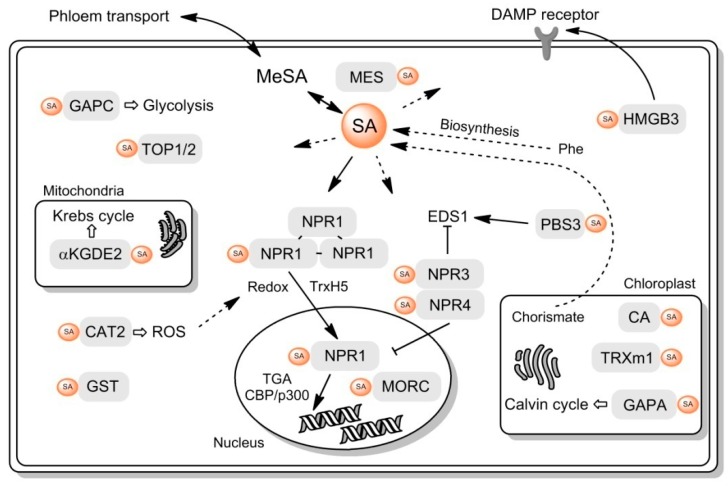
Schematic representation of putative functional roles of SABPs in cell metabolism. Phe, phenylalanine; DAMP, damage-associated molecular pattern; ROS, reactive oxygen species. See the main text for protein abbreviations. Solid arrows represent single enzymatic steps. Dashed arrows represent multiple consecutive enzymatic steps. Hollow arrows represent functional connections to cell activities.

SABPs could play one of the two probable roles: 1.) Act in support to the NPR1 pathway or 2.) Act in parallel to NPR1 (Figure 5). Regarding the first option, the mechanisms of NPR1 monomerization are indeed not fully understood, although we know that they require upstream redox changes to occur. The proteins involved in this process are still a matter of further investigation. Thioredoxins are perfect candidates for disulphide bond reduction in NPR1 oligomers. TRXm1 has been found to be an SABP, however, this protein is in chloroplasts, while cytosolic TRXh5, implicated in NPR1 monomerization, is not a SABP. AtCAT2 is a homolog of tobacco SABP1—both enzymes could also be a part of cell redox regulation (Figure 5). At some point, NPR1 has to be degraded to attenuate the response, and this involves NPR3 and NPR4 [48], but, in addition, TOP1, TOP2 and TPPII—all proteolytic SABPs—could arguably play a part. Another question is what receptor allows the SA-driven activation of PI4K and PLD, which are both linked to NPR1 [40,41,42].

Alternatively, SABPs could act independently of NPR1. Indeed, at least some of the plant reactions to SA occur in an *npr1* genetic background [43,44,45], but our knowledge of the molecules involved in the NPR1-independent pathway is next to none.

At least two enzymes directly involved in energy metabolism have been found to bind SA—GAPDH (glycolysis) and alpha-ketoglutarate dehydrogenase (Krebs cycle). This could be a way for SA to control the stress-to-growth transition of cell metabolism. Alternatively, since cytosolic GAPDH has been shown to be a dual activity enzyme [87], SA could be a trigger for the transition.

SA has an activity in pollen tubes and in isolated organelles. In these systems, the NPR1 pathway is absent. In Arabidopsis, exogenous SA and MeSA had an opposite effect on pollen tube elongation. The inhibitory effect of SA could be due to changes in clathrin-mediated endocytosis. SA inhibited the internalization of FM4-64 dye while this effect was abolished in *chc2-2* (clathrin heavy chain) mutants [45]. MeSA methylesterase- and SA methyltransferase-GFP constructs were both located in growing pollen tips, suggesting that SA is employed in the control of polarized growth.

SA stimulates the activity of mitochondrial succinate dehydrogenase (SDH, respiratory Complex II) and H_2_O_2_ accumulation in isolated mitochondria [100]. The authors concluded that SA could act at the ubiquinone binding site of respiratory Complex II. Since SDH-deficient plants had diminished SA responses and SA-induced ROS production—this can be the bona fide mechanism of SA perception. At the moment, no SABPs have been formally identified to mediate the above-mentioned effects of SA.

Affinity to SA can differ by up to 1000-fold in SABPs (e.g., SABP1 *K*_d_ = 14 μM; NPR4 *K*_d_ = 23 nM). So, at any given moment, based on current cell SA concentration, SA will interact with a limited set of SABPs. In this manner, a regulatory input of SA will differ depending on SA concentration. In Arabidopsis, the basal SA level is around 1 μM, thus some SABPs will bind SA even at basal concentrations, while others will interact only when SA levels rise following stress exposure. The abundance of SABPs (e.g., by transcriptional regulation) is also subjected to regulation (Figure 2).

The uncertainty of a role for SABPs in SA signaling stipulates the need to study SA–protein interaction in planta. In such experiments, the use of isotope-labeled SA is preferred while alternatively photoaffinity labelling [81] could be adopted for protoplast experiments.

Intriguingly, components of the SA-signaling network are still being revealed as we speak. In recent publications, a role for GH3.12/PBS3 was highlighted. PBS3, whose enzymatic activity is regulated by SA [84], was shown to be both an important enzyme of SA biosynthesis [19,20] and signaling (Figure 5), controlling EDS1 [79].

## Supplementary Materials

Supplementary materials can be found at https://www.mdpi.com/1422-0067/20/18/4377/s1.

## References

[1] Dong C.-J., Li L., Shang Q.-M., Liu X.-Y., Zhang Z.-G. (2014). Endogenous salicylic acid accumulation is required for chilling tolerance in cucumber (*Cucumis sativus* L.) seedlings. Planta.

[2] Shin H., Min K., Arora R. (2018). Exogenous salicylic acid improves freezing tolerance of spinach (*Spinacia oleracea* L.) leaves. Cryobiology.

[3] Wang W., Wang X., Huang M., Cai J., Zhou Q., Dai T., Cao W., Jiang D. (2018). Hydrogen Peroxide and Abscisic Acid Mediate Salicylic Acid-Induced Freezing Tolerance in Wheat. Front. Plant. Sci..

[4] Feng B., Zhang C., Chen T., Zhang X., Tao L., Fu G. (2018). Salicylic acid reverses pollen abortion of rice caused by heat stress. BMC Plant Biol..

[5] Moravcová Š., Tůma J., Dučaiová Z.K., Waligórski P., Kula M., Saja D., Słomka A., Bąba W., Libik-Konieczny M. (2018). Influence of salicylic acid pretreatment on seeds germination and some defence mechanisms of Zea mays plants under copper stress. Plant Physiol. Biochem..

[6] Babourina O., Rengel Z., Jayakannan M., Bose J., Shabala S., Poschenrieder C., Massart A. (2015). The NPR1-dependent salicylic acid signalling pathway is pivotal for enhanced salt and oxidative stress tolerance in *Arabidopsis*. J. Exp. Bot..

[7] La V.H., Lee B.-R., Islam M.T., Park S.-H., Jung H.-I., Bae D.-W., Kim T.-H. (2019). Characterization of salicylic acid-mediated modulation of the drought stress responses: Reactive oxygen species, proline, and redox state in *Brassica napus*. Environ. Exp. Bot..

[8] Yang L., Li B., Zheng X.-Y., Li J., Yang M., Dong X., He G., An C., Deng X.W. (2015). Salicylic acid biosynthesis is enhanced and contributes to increased biotrophic pathogen resistance in *Arabidopsis* hybrids. Nat. Commun..

[9] Bernsdorff F., Döring A.-C., Gruner K., Schuck S., Bräutigam A., Zeier J. (2016). Pipecolic Acid Orchestrates Plant Systemic Acquired Resistance and Defense Priming via Salicylic Acid-Dependent and -Independent Pathways. Plant Cell.

[10] Prodhan M.Y., Munemasa S., Nahar M.N.-E.-N., Nakamura Y., Murata Y. (2018). Guard Cell Salicylic Acid Signaling Is Integrated into Abscisic Acid Signaling via the Ca^2+^/CPK-Dependent Pathway. Plant Physiol..

[11] Lee S., Kim S.-G., Park C.-M. (2010). Salicylic acid promotes seed germination under high salinity by modulating antioxidant activity in *Arabidopsis*. New Phytol..

[12] Šašek V., Janda M., Delage E., Puyaubert J., Guivarc’h A., López Maseda E., Dobrev P.I., Caius J., Bóka K., Valentová O. (2014). Constitutive salicylic acid accumulation in pi4kIIIβ1β2 *Arabidopsis* plants stunts rosette but not root growth. New Phytol..

[13] Janda M., Ruelland E. (2015). Magical mystery tour: Salicylic acid signalling. Environ. Exp. Bot..

[14] Nawrath C., Métraux J.-P. (1999). Salicylic Acid Induction-Deficient Mutants of *Arabidopsis* Express PR-2 and PR-5 and Accumulate High Levels of Camalexin after Pathogen Inoculation. Plant Cell.

[15] Wildermuth M.C., Dewdney J., Wu G., Ausubel F.M. (2001). Isochorismate synthase is required to synthesize salicylic acid for plant defence. Nature.

[16] Garcion C., Lohmann A., Lamodière E., Catinot J., Buchala A., Doermann P., Métraux J.-P. (2008). Characterization and biological function of the ISOCHORISMATE SYNTHASE2 gene of *Arabidopsis*. Plant Physiol..

[17] Hao Q., Wang W., Han X., Wu J., Lyu B., Chen F., Caplan A., Li C., Wu J., Wang W. (2018). Isochorismate-based salicylic acid biosynthesis confers basal resistance to *Fusarium graminearum* in barley. Mol. Plant Pathol..

[18] Seguel A., Jelenska J., Herrera-Vásquez A., Marr S.K., Joyce M.B., Gagesch K.R., Shakoor N., Jiang S.-C., Fonseca A., Wildermuth M.C. (2018). PROHIBITIN3 Forms Complexes with ISOCHORISMATE SYNTHASE1 to Regulate Stress-Induced Salicylic Acid Biosynthesis in *Arabidopsis*. Plant Physiol..

[19] Rekhter D., Lüdke D., Ding Y., Feussner K., Zienkiewicz K., Lipka V., Wiermer M., Zhang Y., Feussner I. (2019). Isochorismate-derived biosynthesis of the plant stress hormone salicylic acid. Science.

[20] Torrens-Spence M.P., Bobokalonova A., Carballo V., Glinkerman C.M., Pluskal T., Shen A., Weng J.-K. (2019). PBS3 and EPS1 complete salicylic acid biosynthesis from isochorismate in *Arabidopsis*. bioRxiv.

[21] Huang J., Gu M., Lai Z., Fan B., Shi K., Zhou Y.-H., Yu J.-Q., Chen Z. (2010). Functional analysis of the *Arabidopsis* PAL gene family in plant growth, development, and response to environmental stress. Plant Physiol..

[22] Li Y.-X., Zhang W., Dong H.-X., Liu Z.-Y., Ma J., Zhang X.-Y. (2018). Salicylic acid in *Populus tomentosa* is a remote signalling molecule induced by *Botryosphaeria dothidea* infection. Sci. Rep..

[23] Diaz-Vivancos P., Bernal-Vicente A., Cantabella D., Petri C., Hernández J.A. (2017). Metabolomics and Biochemical Approaches Link Salicylic Acid Biosynthesis to Cyanogenesis in Peach Plants. Plant. Cell Physiol..

[24] Thompson A.M.G., Iancu C.V., Neet K.E., Dean J.V., Choe J.-Y. (2017). Differences in salicylic acid glucose conjugations by UGT74F1 and UGT74F2 from *Arabidopsis thaliana*. Sci. Rep..

[25] Dean J.V., Mohammed L.A., Fitzpatrick T. (2005). The formation, vacuolar localization, and tonoplast transport of salicylic acid glucose conjugates in tobacco cell suspension cultures. Planta.

[26] Zhang K., Halitschke R., Yin C., Liu C.-J., Gan S.-S. (2013). Salicylic acid 3-hydroxylase regulates *Arabidopsis* leaf longevity by mediating salicylic acid catabolism. Proc. Natl. Acad. Sci. USA.

[27] Huang X.-X., Zhu G.-Q., Liu Q., Chen L., Li Y.-J., Hou B.-K. (2018). Modulation of Plant Salicylic Acid-Associated Immune Responses via Glycosylation of Dihydroxybenzoic Acids. Plant Physiol..

[28] Baek D., Pathange P., Chung J.-S., Jiang J., Gao L., Oikawa A., Hirai M.Y., Saito K., Pare P.W., Shi H. (2010). A stress-inducible sulphotransferase sulphonates salicylic acid and confers pathogen resistance in *Arabidopsis*. Plant Cell Environ..

[29] Westfall C.S., Sherp A.M., Zubieta C., Alvarez S., Schraft E., Marcellin R., Ramirez L., Jez J.M. (2016). *Arabidopsis thaliana* GH3.5 acyl acid amido synthetase mediates metabolic crosstalk in auxin and salicylic acid homeostasis. Proc. Natl. Acad. Sci. USA.

[30] Makandar R., Nalam V.J., Chowdhury Z., Sarowar S., Klossner G., Lee H., Burdan D., Trick H.N., Gobbato E., Parker J.E. (2015). The Combined Action of ENHANCED DISEASE SUSCEPTIBILITY1, PHYTOALEXIN DEFICIENT4, and SENESCENCE-ASSOCIATED101 Promotes Salicylic Acid-Mediated Defenses to Limit *Fusarium graminearum* Infection in *Arabidopsis thaliana*. Mol. Plant Microbe Interact..

[31] Bhattacharjee S., Halane M.K., Kim S.H., Gassmann W. (2011). Pathogen Effectors Target *Arabidopsis* EDS1 and Alter Its Interactions with Immune Regulators. Science.

[32] Zhu S., Jeong R.-D., Venugopal S.C., Lapchyk L., Navarre D., Kachroo A., Kachroo P. (2011). SAG101 Forms a Ternary Complex with EDS1 and PAD4 and Is Required for Resistance Signaling against Turnip Crinkle Virus. PLoS Pathog..

[33] Cui H., Qiu J., Zhou Y., Bhandari D.D., Zhao C., Bautor J., Parker J.E. (2018). Antagonism of Transcription Factor MYC2 by EDS1/PAD4 Complexes Bolsters Salicylic Acid Defense in *Arabidopsis* Effector-Triggered Immunity. Mol. Plant.

[34] Li N., Han X., Feng D., Yuan D., Huang L.-J. (2019). Signaling Crosstalk between Salicylic Acid and Ethylene/Jasmonate in Plant Defense: Do We Understand What They Are Whispering?. Int. J. Mol. Sci..

[35] Joglekar S., Suliman M., Bartsch M., Halder V., Maintz J., Bautor J., Zeier J., Parker J.E., Kombrink E. (2018). Chemical Activation of EDS1/PAD4 Signaling Leading to Pathogen Resistance in *Arabidopsis*. Plant Cell Physiol..

[36] Ma J., Chen J., Wang M., Ren Y., Wang S., Lei C., Cheng Z., Sodmergen (2018). Disruption of OsSEC3A increases the content of salicylic acid and induces plant defense responses in rice. J. Exp. Bot..

[37] Wu Y., Zhang D., Chu J.Y., Boyle P., Wang Y., Brindle I.D., De Luca V., Després C. (2012). The *Arabidopsis* NPR1 Protein Is a Receptor for the Plant Defense Hormone Salicylic Acid. Cell Rep..

[38] Mou Z., Fan W., Dong X. (2003). Inducers of Plant Systemic Acquired Resistance Regulate NPR1 Function through Redox Changes. Cell.

[39] Kwon D.-J., Jin H., Kang M.-J., Yun S.-H., Choi S.-M., Noh Y.-S., Noh B. (2018). Salicylic acid-induced transcriptional reprogramming by the HAC–NPR1–TGA histone acetyltransferase complex in *Arabidopsis*. Nucleic Acids Res..

[40] Krinke O., Flemr M., Vergnolle C., Collin S., Renou J.-P., Taconnat L., Yu A., Burketová L., Valentová O., Zachowski A. (2009). Phospholipase D Activation Is an Early Component of the Salicylic Acid Signaling Pathway in *Arabidopsis* Cell Suspensions. Plant Physiol..

[41] Krinke O., Ruelland E., Valentová O., Vergnolle C., Renou J.-P., Taconnat L., Flemr M., Burketová L., Zachowski A. (2007). Phosphatidylinositol 4-Kinase Activation Is an Early Response to Salicylic Acid in *Arabidopsis* Suspension Cells. Plant Physiol..

[42] Janda M., Šašek V., Chmelařová H., Andrejch J., Nováková M., Hajšlová J., Burketová L., Valentová O. (2015). Phospholipase D affects translocation of NPR1 to the nucleus in *Arabidopsis thaliana*. Front. Plant Sci..

[43] Canet J.V., Dobon A., Roig A., Tornero P. (2010). Structure-function analysis of npr1 alleles in *Arabidopsis* reveals a role for its paralogs in the perception of salicylic acid. Plant Cell Environ..

[44] Blanco F., Garretón V., Frey N., Dominguez C., Pérez-Acle T., Van der Straeten D., Jordana X., Holuigue L. (2005). Identification of NPR1-Dependent and Independent Genes Early Induced by Salicylic Acid Treatment in *Arabidopsis*. Plant Mol. Biol..

[45] Rong D., Luo N., Mollet J.C., Liu X., Yang Z. (2016). Salicylic Acid Regulates Pollen Tip Growth through an NPR3/NPR4-Independent Pathway. Mol. Plant..

[46] Castelló M.J., Medina-Puche L., Lamilla J., Tornero P. (2018). NPR1 paralogs of *Arabidopsis* and their role in salicylic acid perception. PLoS ONE.

[47] Moreau M., Tian M., Klessig D.F. (2012). Salicylic acid binds NPR3 and NPR4 to regulate NPR1-dependent defense responses. Cell Res..

[48] Fu Z.Q., Yan S., Saleh A., Wang W., Ruble J., Oka N., Mohan R., Spoel S.H., Tada Y., Zheng N. (2012). NPR3 and NPR4 are receptors for the immune signal salicylic acid in plants. Nature.

[49] Moreau M., Westlake T., Zampogna G., Popescu G., Tian M., Noutsos C., Popescu S. (2013). The *Arabidopsis* oligopeptidases TOP1 and TOP2 are salicylic acid targets that modulate SA-mediated signaling and the immune response. Plant J..

[50] Manohar M., Tian M., Moreau M., Park S.-W., Choi H.W., Fei Z., Friso G., Asif M., Manosalva P., von Dahl C.C. (2015). Identification of multiple salicylic acid-binding proteins using two high throughput screens. Front. Plant Sci..

[51] Klessig D.F., Tian M., Choi H.W. (2016). Multiple Targets of Salicylic Acid and Its Derivatives in Plants and Animals. Front. Immunol..

[52] Chen Z., Klessig D.F. (1991). Identification of a soluble salicylic acid-binding protein that may function in signal transduction in the plant disease-resistance response. Proc. Natl. Acad. Sci. USA.

[53] Chen Z., Ricigliano J.W., Klessig D.F. (1993). Purification and characterization of a soluble salicylic acid-binding protein from tobacco. Proc. Natl. Acad. Sci. USA.

[54] Chen Z., Silva H., Klessig D. (1993). Active oxygen species in the induction of plant systemic acquired resistance by salicylic acid. Science.

[55] Conrath U., Chen Z., Ricigliano J.R., Klessig D.F. (1995). Two inducers of plant defense responses, 2,6-dichloroisonicotinec acid and salicylic acid, inhibit catalase activity in tobacco. Proc. Natl. Acad. Sci. USA.

[56] Yuan H.-M., Liu W.-C., Lu Y.-T. (2017). CATALASE2 Coordinates SA-Mediated Repression of Both Auxin Accumulation and JA Biosynthesis in Plant Defenses. Cell Host Microbe.

[57] Du H., Klessig D.F. (1997). Identification of a Soluble, High-Affinity Salicylic Acid-Binding Protein in Tobacco. Plant Physiol..

[58] Kumar D., Klessig D.F. (2003). High-affinity salicylic acid-binding protein 2 is required for plant innate immunity and has salicylic acid-stimulated lipase activity. Proc. Natl. Acad. Sci. USA.

[59] Forouhar F., Yang Y., Kumar D., Chen Y., Fridman E., Park S.W., Chiang Y., Acton T.B., Montelione G.T., Pichersky E. (2005). Structural and biochemical studies identify tobacco SABP2 as a methyl salicylate esterase and implicate it in plant innate immunity. Proc. Natl. Acad. Sci. USA.

[60] Tripathi D., Jiang Y.-L., Kumar D. (2010). SABP2, a methyl salicylate esterase is required for the systemic acquired resistance induced by acibenzolar-S-methyl in plants. FEBS Lett..

[61] Hartmann M., Zeier T., Bernsdorff F., Reichel-Deland V., Kim D., Hohmann M., Scholten N., Schuck S., Bräutigam A., Hölzel T. (2018). Flavin Monooxygenase-Generated N-Hydroxypipecolic Acid Is a Critical Element of Plant Systemic Immunity. Cell.

[62] Kumar D., Gustafsson C., Klessig D.F. (2006). Validation of RNAi silencing specificity using synthetic genes: salicylic acid-binding protein 2 is required for innate immunity in plants. Plant J..

[63] Park S.-W., Liu P.-P., Forouhar F., Vlot A.C., Tong L., Tietjen K., Klessig D.F. (2009). Use of a synthetic salicylic acid analog to investigate the roles of methyl salicylate and its esterases in plant disease resistance. J. Biol. Chem..

[64] Manosalva P.M., Park S.-W., Forouhar F., Tong L., Fry W.E., Klessig D.F. (2010). Methyl Esterase 1 (StMES1) Is Required for Systemic Acquired Resistance in Potato. Mol. Plant Microbe Interact..

[65] Zhao N., Guan J., Forouhar F., Tschaplinski T.J., Cheng Z.-M., Tong L., Chen F. (2009). Two poplar methyl salicylate esterases display comparable biochemical properties but divergent expression patterns. Phytochemistry.

[66] Vlot A.C., Liu P.-P., Cameron R.K., Park S.-W., Yang Y., Kumar D., Zhou F., Padukkavidana T., Gustafsson C., Pichersky E. (2008). Identification of likely orthologs of tobacco salicylic acid-binding protein 2 and their role in systemic acquired resistance in *Arabidopsis thaliana*. Plant J..

[67] Slaymaker D.H., Navarre D.A., Clark D., del Pozo O., Martin G.B., Klessig D.F. (2002). The tobacco salicylic acid-binding protein 3 (SABP3) is the chloroplast carbonic anhydrase, which exhibits antioxidant activity and plays a role in the hypersensitive defense response. Proc. Natl. Acad. Sci. USA.

[68] DiMario R.J., Clayton H., Mukherjee A., Ludwig M., Moroney J.V. (2017). Plant Carbonic Anhydrases: Structures, Locations, Evolution, and Physiological Roles. Mol. Plant.

[69] Wang Y.-Q., Feechan A., Yun B.-W., Shafiei R., Hofmann A., Taylor P., Xue P., Yang F.-Q., Xie Z.-S., Pallas J.A. (2009). S-Nitrosylation of AtSABP3 Antagonizes the Expression of Plant Immunity. J. Biol. Chem..

[70] Ahmad B., Jaleel H., Sadiq Y., Khan M.M.A., Shabbir A. (2018). Response of exogenous salicylic acid on cadmium induced photosynthetic damage, antioxidant metabolism and essential oil production in peppermint. Plant Growth Regul..

[71] Medina-Puche L., Castelló M.J., Canet J.V., Lamilla J., Colombo M.L., Tornero P. (2017). β-carbonic anhydrases play a role in salicylic acid perception in *Arabidopsis*. PLoS ONE.

[72] Poque S., Wu H.-W., Huang C.-H., Cheng H.-W., Hu W.-C., Yang J.-Y., Wang D., Yeh S.-D. (2017). Potyviral gene-silencing suppressor HCPro interacts with salicylic acid (SA)-binding protein 3 to weaken SA-mediated defense responses. Mol. Plant Microbe Interact..

[73] Tada Y., Spoel S.H., Pajerowska-Mukhtar K., Mou Z., Song J., Wang C., Zuo J., Dong X. (2008). Plant Immunity Requires Conformational Charges of NPR1 via S-Nitrosylation and Thioredoxins. Science.

[74] Geigenberger P., Thormählen I., Daloso D.M., Fernie A.R. (2017). The Unprecedented Versatility of the Plant Thioredoxin System. Trends Plant Sci..

[75] Ruelland E., Miginiac-Maslow M. (1999). Regulation of chloroplast enzyme activities by thioredoxins: Activation or relief from inhibition?. Trends Plant Sci..

[76] Ding Y., Sun T., Ao K., Peng Y., Zhang Y., Li X., Zhang Y. (2018). Opposite Roles of Salicylic Acid Receptors NPR1 and NPR3/NPR4 in Transcriptional Regulation of Plant Immunity. Cell.

[77] Zhang Y., Cheng Y.T., Qu N., Zhao Q., Bi D., Li X. (2006). Negative regulation of defense responses in *Arabidopsis* by two NPR1 paralogs. Plant. J..

[78] Liu G., Holub E.B., Alonso J.M., Ecker J.R., Fobert P.R. (2005). An *Arabidopsis* NPR1-like gene, NPR4, is required for disease resistance. Plant. J..

[79] Chang M., Zhao J., Chen H., Li G., Chen J., Li M., Palmer I.A., Song J., Alfano J.R., Liu F. (2019). PBS3 Protects EDS1 from Proteasome-Mediated Degradation in Plant Immunity. Mol. Plant.

[80] Zhou X.-T., Jia L.-J., Wang H.-Y., Zhao P., Wang W.-Y., Liu N., Song S.-W., Wu Y., Su L., Zhang J. (2018). The potato transcription factor StbZIP61 regulates dynamic biosynthesis of salicylic acid in defense against *Phytophthora infestans* infection. Plant. J..

[81] Tian M., von Dahl C.C., Liu P.-P., Friso G., van Wijk K.J., Klessig D.F. (2012). The combined use of photoaffinity labeling and surface plasmon resonance-based technology identifies multiple salicylic acid-binding proteins. Plant J..

[82] Gullner G., Komives T., Király L., Schröder P. (2018). Glutathione S-Transferase Enzymes in Plant-Pathogen Interactions. Front. Plant Sci..

[83] Westfall C.S., Zubieta C., Herrmann J., Kapp U., Nanao M.H., Jez J.M. (2012). Structural Basis for Prereceptor Modulation of Plant Hormones by GH3 Proteins. Science.

[84] Okrent R.A., Brooks M.D., Wildermuth M.C. (2009). *Arabidopsis* GH3.12 (PBS3) conjugates amino acids to 4-substituted benzoates and is inhibited by salicylate. J. Biol. Chem..

[85] Schuman M.C., Meldau S., Gaquerel E., Diezel C., McGale E., Greenfield S., Baldwin I.T. (2018). The Active Jasmonate JA-Ile Regulates a Specific Subset of Plant Jasmonate-Mediated Resistance to Herbivores in Nature. Front. Plant Sci..

[86] Nobuta K., Okrent R.A., Stoutemyer M., Rodibaugh N., Kempema L., Wildermuth M.C., Innes R.W. (2007). The GH3 acyl adenylase family member PBS3 regulates salicylic acid-dependent defense responses in Arabidopsis. Plant Physiol..

[87] Tian M., Sasvari Z., Gonzalez P.A., Friso G., Rowland E., Liu X.-M., van Wijk K.J., Nagy P.D., Klessig D.F. (2015). Salicylic Acid Inhibits the Replication of Tomato bushy stunt virus by Directly Targeting a Host Component in the Replication Complex. Mol. Plant Microbe Interact..

[88] Choi H.W., Tian M., Manohar M., Harraz M.M., Park S.-W., Schroeder F.C., Snyder S.H., Klessig D.F. (2015). Human GAPDH Is a Target of Aspirin’s Primary Metabolite Salicylic Acid and Its Derivatives. PLoS ONE.

[89] Liao Y., Tian M., Zhang H., Li X., Wang Y., Xia X., Zhou J., Zhou Y., Yu J., Shi K. (2015). Salicylic acid binding of mitochondrial alpha-ketoglutarate dehydrogenase E2 affects mitochondrial oxidative phosphorylation and electron transport chain components and plays a role in basal defense against tobacco mosaic virus in tomato. New Phytol..

[90] Wang R., Rajagopalan K., Sadre-Bazzaz K., Moreau M., Klessig D.F., Tong L. (2014). Structure of the *Arabidopsis thaliana* TOP2 oligopeptidase. Acta Crystallogr. Sect. F Struct. Biol. Commun..

[91] Westlake T.J., Ricci W.A., Popescu G.V., Popescu S.C. (2015). Dimerization and thiol sensitivity of the salicylic acid binding thimet oligopeptidases TOP1 and TOP2 define their functions in redox-sensitive cellular pathways. Front. Plant Sci..

[92] Moissiard G., Bischof S., Husmann D., Pastor W.A., Hale C.J., Yen L., Stroud H., Papikian A., Vashisht A.A., Wohlschlegel J.A. (2014). Transcriptional gene silencing by *Arabidopsis* microrchidia homologues involves the formation of heteromers. Proc. Natl. Acad. Sci. USA.

[93] Manohar M., Choi H.W., Manosalva P., Austin C.A., Peters J.E., Klessig D.F. (2017). Plant and Human MORC Proteins Have DNA-Modifying Activities Similar to Type II Topoisomerases, but Require One or More Additional Factors for Full Activity. Mol. Plant Microbe Interact..

[94] Choi H.W., Manohar M., Manosalva P., Tian M., Moreau M., Klessig D.F. (2016). Activation of Plant Innate Immunity by Extracellular High Mobility Group Box 3 and Its Inhibition by Salicylic Acid. PLoS Pathog..

[95] Hruz T., Laule O., Szabo G., Wessendorp F., Bleuler S., Oertle L., Widmayer P., Gruissem W., Zimmermann P. (2008). Genevestigator V3: A Reference Expression Database for the Meta-Analysis of Transcriptomes. Adv. Bioinform..

[96] Barber R.D., Harmer D.W., Coleman R.A., Clark B.J. (2005). GAPDH as a housekeeping gene: analysis of GAPDH mRNA expression in a panel of 72 human tissues. Physiol. Genom..

[97] Pettersen E.F., Goddard T.D., Huang C.C., Couch G.S., Greenblatt D.M., Meng E.C., Ferrin T.E. (2004). UCSF Chimera—A visualization system for exploratory research and analysis. J. Comput. Chem..

[98] Gupta V., Liu S., Ando H., Ishii R., Tateno S., Kaneko Y., Yugami M., Sakamoto S., Yamaguchi Y., Nureki O. (2013). Salicylic Acid Induces Mitochondrial Injury by Inhibiting Ferrochelatase Heme Biosynthesis Activity. Mol. Pharmacol..

[99] Delaney T.P., Friedrich L., Ryals J.A. (1995). *Arabidopsis* signal transduction mutant defective in chemically and biologically induced disease resistance. Proc. Natl. Acad. Sci. USA.

[100] Belt K., Huang S., Thatcher L.F., Casarotto H., Singh K.B., Van Aken O., Millar A.H. (2017). Salicylic Acid-Dependent Plant Stress Signaling via Mitochondrial Succinate Dehydrogenase. Plant Physiol..

