# Supplementary material for: Salicylic Acid Binding Proteins (SABPs): The Hidden Forefront of Salicylic Acid Signalling
Source: Int J Mol Sci. 2019 Sep 6;20(18):4377. doi: 10.3390/ijms20184377 (PMC6769663; doi:10.3390/ijms20184377)
Supplement: Supplementary file 1 [file ijms-20-04377-s001.pdf]

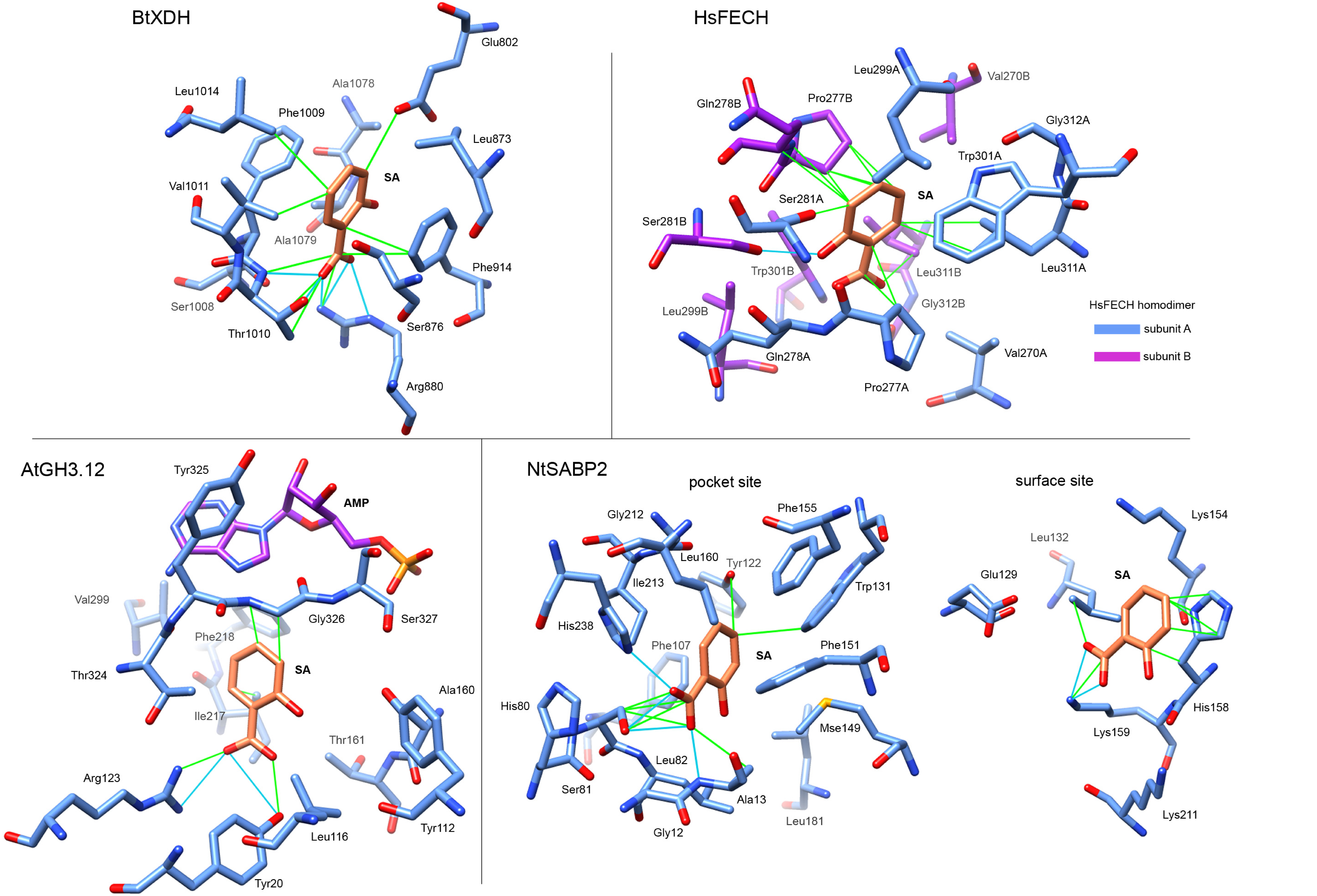

Figure S1. Amino acid residues in direct vicinity (≤5Å) of a SA ligand. Ligand interactions were visualized using the UCSF Chimera (https://www.cgl.ucsf.edu/chimera/). Hydrogen bonds are depicted as blue lines; other bonds (cumulatively polar and non-polar) are depicted as green lines. At, *A. thaliana*; Bt, *Bos taurus*; FECH, ferrochelatase; GH3.12, Gretchen Hagen 3.12; Hs, *Homo sapience*; Mse, selenomethionine; Nt, *Nicotiana tabacum*; SABP, salicylic acid binding protein; XDH, xanthine dehydrogenase.
